# Disclosure of HIV Status to Children in Sub-Saharan Africa: A Systematic Review

**DOI:** 10.3390/medicina55080433

**Published:** 2019-08-02

**Authors:** Abdul-Razak Doat, Reza Negarandeh, Marzieh Hasanpour

**Affiliations:** 1 School of Nursing and Midwifery, Tehran University of Medical Sciences, Tehran 11369, Iran; 2 Nursing and Midwifery Care Research Center, School of Nursing and Midwifery, Tehran University of Medical Sciences, Tehran 11369, Iran; 3 NIDCAP Professional, Neonatal Intensive Care and Pediatric Nursing Education Department, School of Nursing and Midwifery, Tehran University of Medical Sciences, Tehran 11369, Iran

**Keywords:** HIV, disclosure, sub-Saharan Africa, children

## Abstract

*Background and objectives:* This study aimed to assess the level of HIV disclosure to children in sub-Saharan Africa as it relates to prevalence of disclosure, barriers, merits and demerits, timing of disclosure, and factors that promote parents and caregivers’ decisions to disclose the information. *Materials and Methods:* A systematic literature search was performed using the following online databases: PubMed, Google Scholar, Web of Science, Scopus, and Embase, to obtain relevant articles on HIV disclosure to children in sub-Saharan Africa. The following search terms were used: “HIV” AND “Disclosure” AND “Sub-Saharan Africa” AND “Children”. *Results:* A total of 18 articles were included in this systematic review. The studies on HIV status disclosure to children in sub-Saharan Africa included a total of 1343 HIV-positive children and 1879 caregiver/child or healthcare worker-child dyads, from the following countries: Ethiopia, South Africa, Ghana, Kenya, Cote d’Ivoire, Burundi, Cameroon, Democratic Republic of Congo, Uganda, Burkina Faso, and Zambia. The prevalence of HIV disclosure ranged from as low as 9% to 72%. Age was a major factor associated with disclosure. *Conclusions:* HIV status disclosure to children is quite low in sub-Saharan Africa. This is a result of multiple factors such as parents’/caregivers’ fear of the child disclosing status to others, a lack of knowledge on how the disclosure should be made, and the assertion that the children are young and cannot withstand the psychological impact of diagnosis.

## 1. Introduction

The global burden of children infected with HIV is significantly very high, with approximately 1.8 million children under the age of 15 years living with the disease worldwide. In addition, 83% of those living with HIV in sub-Saharan Africa are children [1]. While great strides have been made in preventing mother-to-child transmission of HIV, about 150,000 children acquired HIV in 2015 [1]. Mother-to-child transmission (MTCT) is the main mode of HIV transmission in children under the age of 15. The prevalence is also higher in sub-Saharan African countries [2]. The advent of antiretroviral therapy (ART) has allowed prenatally HIV-infected children to grow beyond adolescence and into adulthood [3].

The increased survival rate of children infected with HIV has made HIV status disclosure very important. Disclosure is of clinical importance in children as it will help them comply with the HIV treatment regimen [4]. Children who are informed of their HIV status have higher self-esteem than their colleagues who are unaware of their status. The depression rate is reduced in parents who are able to disclose the HIV status of their children to them as compared to parents who decide to keep it to themselves [5]. Discussing HIV diagnosis with children is complex, as it is filled with many issues such as parents’ guilt, the impact on the child’s emotional health, the reaction of children, a poor understanding of the psychosocial needs and cognitive abilities of children, questions the child may ask their parents about the condition, and the fear of accidental disclosure to other people [6].

The success of ART in pediatric HIV treatment has transformed the epidemic in children in resource-constrained settings, enabling them to live much longer then what used to be the norm [7]. There is therefore the need for a change in the practice of nondisclosure of HIV status to children [8]. The World Health Organization (WHO) recommendation spells out that children should be made aware of their HIV-positive status between the ages of 10 and 12. This is often not the case in most parts of sub-Saharan Africa, though [9]. Additionally, there is a disclosure manual developed by the WHO for healthcare workers on how to support children up to 12 years of age and their caregivers with disclosure of HIV status [10]. It is largely underutilized in sub-Saharan Africa, where caregivers are often reluctant to inform children of their HIV diagnosis because of concerns regarding the child inadvertently revealing the family’s HIV status to others [11]. This systematic review aims to assess the level of HIV disclosure to children in sub-Saharan Africa as it relates to prevalence of disclosure to children, barriers, and merits and demerits of disclosure. Timing of disclosure and factors that promote parents’ and caregivers’ decisions to disclose the child’s HIV status will be examined as well.

## 2. Materials and Methods

We conducted this systematic review in accordance with the Preferred Reporting Items for Systematic Reviews and Meta-Analyses (PRISMA) recommendations [12].

### 2.1. Search Strategy

A systematic literature search was performed using the following online databases: PubMed, Google Scholar, Web of Science, Scopus, and Embase. A core strategy was developed in PubMed and then translated for other databases. The following search terms were used: “HIV” AND “Disclosure” AND “Sub-Saharan Africa” AND “Children”. Furthermore, we manually screened the references of retrieved studies to obtain related articles. Afterwards, all retrieved studies were entered into Endnote software version X7 (Thomson Reuters, Philadelphia, PA, USA) to manage citations as well as for the identification of duplicate articles.

### 2.2. Inclusion Criteria

Studies were included if they met the following criteria:

(i) The study population was children living with HIV in sub-Saharan Africa.
(ii) The study investigated HIV disclosure status in children in sub-Saharan Africa.

### 2.3. Exclusion Criteria

Articles that were not published in the English language, review articles and letters, studies that did not clearly present children’s HIV disclosure data, studies that were not about HIV/AIDS, and studies that were not related to sub-Saharan Africa were excluded.

### 2.4. Data Extraction

Two independent authors (A.R.D. and R.N.) independently assessed the studies for eligibility. Studies that met the inclusion criteria were evaluated and included. Disagreements were resolved through consensus, and in cases where there was no consensus, discussion with a third party (M.H.) was used to address it. The following data were extracted from the included studies: first author (year), location, study design, sample description, and key findings.

## 3. Results

### 3.1. Search Results

**Figure 1:**
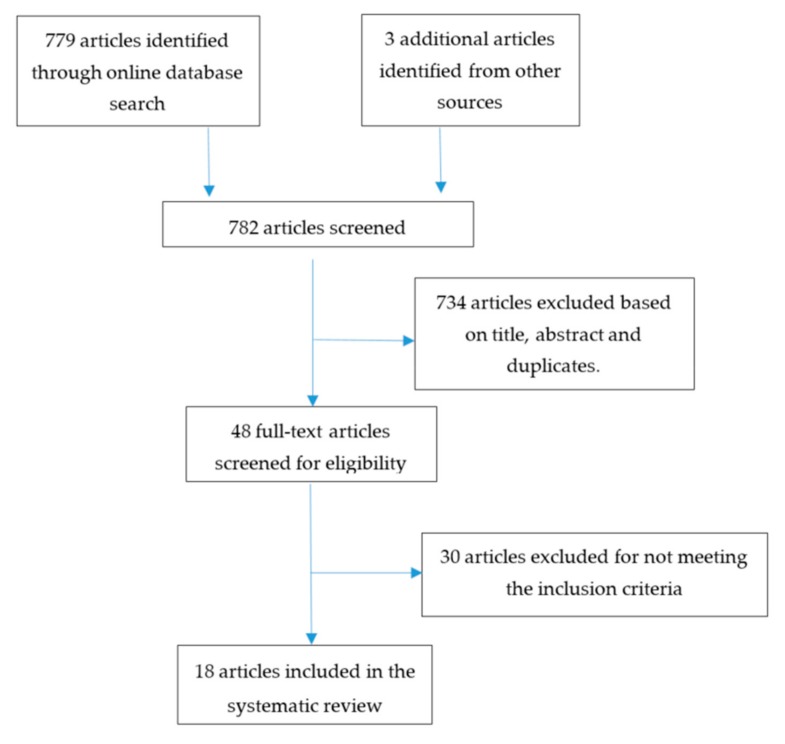
PRISMA flow diagram showing the process of our literature search.

Figure 1 gives the systematic breakdown of our literature search. Our initial search results yielded 779 articles from the various databases. Manual referencing identified three additional articles. After applying the inclusion and exclusion criteria as well as the removal of duplicate articles, 764 articles were excluded. A total of 18 articles were included in this systematic review.

The studies on HIV status disclosure to children in sub-Saharan Africa included a total of 1343 HIV-positive children and 1879 caregiver/child or healthcare worker/child dyads, from the following countries: Ethiopia, South Africa, Ghana, Kenya, Cote d’Ivoire, Burundi, Cameroon, Democratic Republic of Congo, Uganda, Burkina Faso, and Zambia. Furthermore, 12 of these studies were quantitative [7,13,14,15,16,17,18,19,20,21,22,23], while six were qualitative [9,24,25,26,27,28].

The findings from these studies as regards HIV status disclosure to children are summarized below.

### 3.2. Barriers to HIV Disclosure

From the included studies, the following were given as barriers to disclosure:

**I.**  **Age**

It was observed in 39% of the included studies [14,15,16,18,19,24,27] that the age of the child has an influence on HIV disclosure. In a study by Alemu et al., parents were of the opinion that the child would not understand the consequences of HIV diagnosis [14]. This was corroborated by studies by Madiba et al. [15], Kallem et al. [19], Paintsil et al. [23], and Mburu et al. [27].

**II.**  **Psychological Effects**

This was reported in [7,9,13,14,16,19,24,25]. Due to the psychological implications of disclosure to the child, caregivers were reluctant to reveal the child’s HIV status. It was their own way of protecting the child from harm [16], negative reactions [7,25], and psychological disturbances [9,19,24]. In a study by John Stewart et al. involving already-hospitalized children, parents’ reasons for nondisclosure hinged on their fears that knowledge of HIV status might worsen the child’s condition [18].

**III.**  **Child’s Inability to Keep Diagnosis Secret**

The inability of children to keep a diagnosis to themselves was given as a reason for nondisclosure in several studies [7,15,16,19,25,26]. This was also attributed to the child’s age. Caregivers felt that, due to the vulnerability of the child at that age, they might openly discuss their HIV status with peers, friends, schoolmates or the community at large.

**IV.**  **Stigma**

It is well known that HIV patients are discriminated against and stigmatized in society. As a result, caregivers would not want their children to be faced with these negative societal reactions. Hence, the reason for nondisclosure to the children, as observed in the following studies [7,9,14,16,23,24,25,26,27]. Heeren et al. observed that the ripple effect of stigmatization of the child could lead to isolation, fear of blame, guilt, and gossip [25]. Murnane et al. observed that another adult living in the same household as the caregiver and the child who was unaware of his or her status made it more challenging for the caregiver to disclose disease status to the child for fear of the child spreading the news, which might lead to stigmatization [21].

**V.**  **Insufficient Knowledge of the Caregivers**

The following studies [7,9,14,23,25] reported that insufficient knowledge and skills among caregivers were a hindrance to disclosure. It was observed that most of the caregivers had limited knowledge and expertise on how to disclose HIV status to a child [7]. Paintsil et al. pointed out that the literacy level of the caregiver was another factor. Caregivers who had a higher level of education were more likely to reveal the child’s status [23].

**VI.**  **Norms**

In sub-Saharan Africa, open sex education is usually perceived as a taboo. Hence, it is not common to find parents discussing this aspect with their children. Hayfrom-Benjamin et al. observed that, because caregivers are not open to discuss sex with the child, for fear of being asked how they got infected, they might decide not to disclose the child’s status [7]. This was also observed by Mburu et al. [27].

### 3.3. Factors that Promote Disclosure

From the included studies, the following were given as reasons for HIV disclosure to children.

**I.**  **Increasing Age**

It was observed that the advancing age of the child was a key motivation for caregivers to disclose disease status to him or her. Most caregivers preferred to disclose the HIV status to older children because they believed they would understand the nature of the diagnosis and keep it secret [15]. Murnane et al. observed that the number of children whose status had been disclosed to them increased with increasing age [21]. This was corroborated by Meless et al. [17] and Alemu et al. [14].

**II.**  **On ART**

Being on ART was observed as a reason for disclosure of the child’s status. This was observed in [14,17,19]. Alemu et al. observed that disclosure status in children increased with the length of time on ART [14]. This is supported by Meless et al., who observed that HIV disclosure was significantly higher in adolescents with a history of ART treatment [17].

**III.**  **Child’s Right to Know**

The child’s right to knowledge about their HIV status was observed by the following studies [15,25] as a reason for disclosing to children. Madiba et al. observed that most caregivers chose to disclose the child’s status because they felt it was the right of the child to know his or her status [15]. This assertion was corroborated by Heeren et al. [25].

**IV.**  **To Promote Adherence**

Promoting adherence to HIV treatment was also cited as a main motivation for caregivers to disclose the child’s status to them. This was observed in [15,18,19,22]. Since the child is to stay on the ART medication for the rest of their lives, caregivers believe that to promote adherence to treatment it is prudent to disclose the child’s status to them with the hope that the child will adhere to the medication regimen [15]. This notion was also supported by John-Stewart et al. [18] and Montalto et al. [20]. On the contrary, Kallem et al. observed that the lack of adherence to ART was a motivation for disclosure of the child’s status [19]. Though caregivers’ reason for disclosure of HIV status to children was to promote adherence, Newman et al. observed that there was no association between disclosure status and ART adherence [22].

**V.**  **Child Asking Questions**

The child’s quest to know why he or she is constantly taking medication or falling sick by posing questions to caregivers was part of the reason why some caregivers opted to disclose the HIV status to the child. Some studies [9,18] observed that a child asking questions or being curious was a motivating factor for HIV status disclosure. In the study by Namukwaya et al., a caregiver describes how a child kept asking question about his illness and constant intake of medication as he was growing up [9]. Acts of this nature prompt caregivers to disclose a child’s status.

**VI.**  **Other Factors**

There were other factors associated with disclosure in some of the studies. The religion of the caregiver, the caregiver being a family member [14], the frail health of the child [9,18], the level of education of the child, the death of the biological father [19], improved immunological status [20], someone in the child’s school having knowledge of his or her status [21], an adherence crisis, and the importance of medication [9] were among the factors cited.

### 3.4. Prevalence of Disclosure

The prevalence of HIV status disclosure to children was reported in 61% of the included studies [7,13,14,15,16,17,18,19,21,22,24]. The prevalence ranged from as low as 9% in the study by Murnane et al. [21] to as high as 72% in that of Dusabe-Richards et al. [24]. Age was a strong predictor of full disclosure and, as such, the rate of disclosure increased for older children.

### 3.5. Timing of Disclosure

Timing of HIV disclosure is an important component in the management of children living with the disease; 39% of the included studies [13,14,15,19,24,25,28] reported on it. Abebe et al. observed that most caregivers delayed disclosure until children were much older because some of them believed the children were too young to understand, would share the information with others, and would suffer negative emotional consequences at a younger age. The majority of caregivers agreed that HIV status should be disclosed to children much later on, between the ages of six and 20 [13]. This was corroborated by Madiba et al. [15], Kallem et al. [19], and Heeren et al. [25]. Alemu et al. also observed that most caregivers preferred to delay the disclosure until the child was 10 or older [14]. This assertion was also observed by Myer et al. [28].

### 3.6. Merits and Demerits of HIV Disclosure

It was observed that 22% of the included studies [9,24,26,27] reported on the merits and demerits of HIV status disclosure to children.

**I.**  **Merits**

Dusabe-Richards et al. observed that children whose status was disclosed to them took control of their healthcare, visiting a health facility unaccompanied and ensuring their medications were taken regularly and on time, communicated freely about their physical health, and were able to ask questions and access help from caregivers [24]. Mburu et al. observed that disclosure created opportunities for adolescents to access adherence support and other forms of psychosocial support from family members and peers [27]. Fabienne Hejoaka also observed that HIV status disclosure to children helped them to develop strategies to conceal the medicines and the disease [26].

**II.**  **Demerits**

Mburu et al. observed that disclosure of HIV status led to children becoming anxious, depressed, and blaming themselves, and occasionally strained adolescents’ sexual relationships [27]. Namukwaya et al. observed that disclosure of HIV status to children led to them being terrified, worried, confused, and intensely emotional [9].

Summaries of the studies included are presented in Table 1 and Table 2.

## 4. Discussion

This systematic review has examined the pediatric HIV population in sub-Saharan Africa, specifically the disclosure of their HIV status. These were findings from caregivers/parents as well as children living with the infection. The results showed that barriers to disclosure were mostly due to age, the expected psychological effect on the child, knowledge, a child’s inability to keep their HIV status secret, fear of stigmatization, and level of education/literacy. These factors were largely related to the child’s age. Furthermore, we assessed the prevalence of disclosure, which ranged between 9% and 72%. The merits and demerits were also assessed.

Parents’/caregivers’ decision to disclose or not disclose children’s HIV status hinged on the age of the child. Caregivers feared the psychological implications of disclosing HIV status to children, and worried that young children might reveal the diagnosis to others, which could lead to unwarranted discrimination. As such, the prevalence of disclosure to younger children was much lower than that to older children in this present study. Furthermore, caregivers believed that delaying disclosure until the child was much older would help the child accept the diagnosis and keep it a secret. This assertion is corroborated by a study by Oberdorfer et al., in which it was observed that caregivers preferred to delay disclosure because they feared it would have negative emotional consequences for the children and that the child might not be able to keep it a secret, but they were ready to disclose the child’s status when the child was much older [29]. Kouyoumdjian et al. also observed that caregivers were concerned that if the child knew his/her HIV status, s/he might tell other people in the community, which would lead to discrimination towards the child and family [30].

HIV status disclosure to children has its merits as well as demerits, as seen in this review. Most of the findings reported that children whose status was disclosed to them took charge of their own care. There was an improvement in medication adherence and a reduced risk of transmission of HIV to others, and they could communicate freely about their health and seek help from caregivers when necessary. This assertion is supported by the work of Vreeman et al., whose findings revealed that HIV status disclosure to children improved ART adherence, children’s well-being, and their social relationships [4]. They also developed strategies to conceal their status from persons whom they felt should not know about it. Disclosure also brought relief to caregivers/parents, who often had to provide alternative, misleading answers to questions posed to them by the affected children. Nonetheless, disclosure also had a negative effect on children who got to know their status either through caregivers/parents or accidentally through healthcare providers or other family members. Some were furious for a time, while some felt anxious and others became depressed. This is in agreement with the findings of Okawa et al. [31].

There are some limitations to this present review: we did not include unpublished studies, and only articles written in the English language were used. No study was excluded with regards to methodological rigor because there are few studies on HIV disclosure conducted in sub-Saharan Africa. Most of the included quantitative studies used cross-sectional design, describing determinants of disclosure. There is the need for more longitudinal studies as well as more qualitative studies for researchers to evaluate the impact of HIV disclosure over time in this population. However, the study findings contribute to the body of knowledge on HIV disclosure to children in sub-Saharan Africa.

## 5. Conclusions

In this study it is evident that HIV status disclosure to children is quite low in sub-Saharan Africa. This is as a result of multiple factors such as parents’/caregivers’ fear of the child disclosing his/her HIV status to others, a lack of knowledge on how the disclosure should be made, and an assertion that children are too young to withstand the psychological impact of a diagnosis. It is imperative for healthcare providers and caregivers/parents to be trained in how to make age-appropriate disclosures to children. It is advisable for the various health agencies in the region to make guidelines on HIV disclosure available to healthcare providers to make it much easier to deliver age-appropriate information. There is a need for more awareness creation, as evidence from this study shows that the merits of disclosure to children far outweigh the demerits. Support services should be made available for children who become traumatized because of the disclosure of their HIV status. 

## Figures and Tables

**Table 1:** Summary of quantitative studies.

First Author (Year)	Location	Study Design	Sample Description	Key Findings
W. Abebe and S. Teferra, (2012)	Addis Ababa, Ethiopia	Cross-sectional survey	172 parents/caregivers HIV-infected children	Only 16.3% of HIV-infected school children knew their diagnosis. Child’s age was the main predictor of disclosure; the main reason for nondisclosure was fear of negative emotional consequences for the child.
Alemu et al. (2013)	Bahir Dar, Ethiopia	Cross-sectional study	231 caregivers of HIV-positive children	Prevalence of disclosure of children’s HIV-positive status was 31.5%. Religion of caregivers, number of family members, age of child, child’s age when ART started, and time on ART were found to have a statistically significant association with disclosure of HIV-positive status to HIV-infected children.
Madiba & Mokgatle (2017)	Mpumalanga province, South Africa	Cross-sectional survey	405 caregivers of perinatally HIV infected children	Prevalence of disclosure was 27% .26% stated that disclosure was done to promote adherence, while 43% indicated that it was the child’s right to know his/her status. Child’s age was significantly associated with disclosure. 74.5% of caregivers stated that nondisclosure was because children were too young and would not understand the implications of HIV diagnosis and 7% believed the child would not keep the diagnosis secret. 32% of caregivers substituted HIV with other, less stigmatizing conditions in response to children’s questions.
Gyamfi et al. (2017)	Lower Manya Krobo District, Ghana	Cross-sectional study	118 caregivers of HIV-infected children	66.7% of caregivers had not disclosed the HIV status to children infected with the disease. Main barriers to disclosure included age of child, perceived cause of HIV, stigma attached to HIV, child’s inability to keep diagnosis to self, and fear of psychological harm to child.
Hayfron-Benjamin et al. (2018)	Central Region, Ghana	Quantitative analytical survey	103 family caregivers of HIV-infected children	23.3% of caregivers disclosed HIV status to infected children. Barriers to disclosure were the caregiver’s lack of knowledge regarding the disclosure process and when to disclose, the fear of the child’s reaction, and fear of stigmatization and associated negative social consequences.
John-Stewart et al. (2013)	Nairobi, Kenya	Cross-sectional survey	271 caregiver–child dyads	Prevalence of disclosure to the child was only 19%. 79% of caregivers believed children should know their HIV status. Reasons for disclosure included medication adherence, curiosity or illness, while reasons for nondisclosure included age and fear of inadvertent disclosure.
Kallem et al. (2010)	Accra, Ghana	Cross-sectional study	71 caregiver–child dyads	Prevalence of disclosure was 21%. Age of child, level of education of child, deceased biological father, taking own HIV medications, and longer duration on HIV medication were significantly associated with disclosure.
Murnane et al. (2016)	Johannesburg, South Africa	Cohort study	553 perinatally HIV-infected children	9% of children living with HIV had received full disclosure. Prevalence of full disclosure increased with age, from 0% at 4 years of age to 4% at 5 years, 8% at 6 years, and 13%, 16%, and 15% at 7, 8, and 9 years. Age was the strongest predictor of full disclosure and knowing that someone at the child’s school was aware of child’s status was associated with an increased probability of disclosure. A reduced probability of disclosure was related to an adult living in the household who was unaware of the child’s status.
Meless et al. (2013)	Abidjan, Cote d’Ivoire	Cross-sectional study	229 adolescents living with HIV	Out of 193 patients who had HIV documentation, 32.6% were informed of their HIV status. Disclosure status increased significantly with age: 19% for 13–15 years, 33% for 16–18 years, and 86% for 19–21 years. Factors that promoted disclosure included age and being on ART.
Montalto et al. (2017)	Kericho, Kenya	Retrospective, longitudinal studies	96 ALHIV	84 patients improved with disclosure, from a mean of 0.802 pre-disclosure to 0.917 post-disclosure (*p* = 0.0015). ART adherence and improved immunological status are both associated with disclosure of HIV infection to adolescent patients.
Paintsil et al. (2015)	Accra & Kumasi, Ghana	RCT	298 caregivers	The findings did not report on prevalence, but the barriers associated with HIV nondisclosure were the level of education of caregivers, health literacy, and HIV-associated stigma.
Newman et al. (2016)	Burundi, Cameroon, and Democratic Republic of Congo	Cohort study	290 HIV-positive children	144, representing 49.6% of children living with HIV, knew their status. Of the 144, 33% learned of their serostatus at 5–11 years old, 26% at 12+ years, and 13% at younger than five. Differences were observed in length of time on ART: 11% of children who knew their HIV status and 30% of those who did not had been on ART for more than 24 months (*p* < 0.001).

**Table 2:** Summary of qualitative studies.

First Author (Year)	Location	Study Design	Sample Description	Key Findings
Richards et al. (2016)	Uganda	Interviews were conducted for 10 males and 8 female HIV-positive children, as well as 4 male and 14 female caregivers.	18 HIV-positive children and their older caregivers.	72.2% of the HIV-positive children knew their status. Caregivers who had not disclosed the HIV status to the child in their care described feeling fearful for the psychological well-being of the child. Children often described “feeling bad” when they were first told about their HIV-positive status. Disclosure allowed children to take control of their healthcare by proactively seeking care when necessary, visiting a healthcare facility unaccompanied, and ensuring that their medication was taken regularly and on time. They could communicate effectively about their physical health and were able to ask questions and access help from their caregivers.
Namukwaya et al. (2013)	Kampala, Uganda	In-depth interviews	16 care dyads or caregivers and 26 HIV-positive young people	A key impediment to disclosure was that caregivers feared that it would damage their relationships with the young people. Young people did not report prolonged feelings of blame or anger toward their caregivers about their own infection, but they did express frustration at the delay and obfuscation surrounding the disclosure process.
Heeren et al. (2012)	Eastern Cape Province, South Africa	Focus group discussion	51 caregivers, 24 healthcare providers and 5 HIV-positive children	Study participants believed that children should begin to learn about their illness from age five, with full disclosure by age 12. They also believed that the primary caregiver was the best person to disclose the HIV status to a child. They suggested that many caregivers fail to fully inform their children because of (a) lack of knowledge about HIV and its treatment; (b) concern that the children might react negatively; and (c) fear that the children might reveal the information to others, which would occasion gossip, stigmatization, and discrimination toward them and their family.
Hejoaka et al. (2009)	Burkina Faso	Participant observation and semi-structured interview	20 women mothering HIV-positive children & 15 children infected with HIV	In daily care mothers face many great challenges, ranging from the routine of pill-taking to disturbing discussions with children asking questions about their health or treatment. The results also show how HIV/AIDS-related stigma adds an additional layer to the burden of care, compelling mothers to deal with the tension between secrecy surrounding the disease and the openness required in providing care and receiving social support. As mothers live in fear of disclosure, they have to develop concealment strategies around children’s treatment and the nature of the disease.
Mburu et al. (2014)	Kalomo, Kitwe, & Lusaka, Zambia	Interviews & focus group discussion	58, and 53 adolescent living with HIV participated in interview and focus group discussion respectively. 14, and 24 health workers participated in interview and focus group discussion respectively, while 21 parents participated in focus group discussion	The study identified three main barriers to disclosure of HIV status: local norms that deter parents from communicating with their children about sexuality; fear of HIV stigma; and an underlying presumption that adolescents would not understand the consequences of a HIV diagnosis for their lives and relationships. Disclosure had various outcomes at the individual and interpersonal levels. At the individual level, some adolescents described being anxious, depressed, and blaming themselves after being told they had HIV. At the interpersonal level, disclosure created opportunities for adolescents to access adherence support and other forms of psychosocial support from family members and peers. At the same time, it occasionally strained adolescents’ sexual relationships, although it did not always lead to rejection.
Myer et al. (2006)	Cape Town, South Africa	Semi-structured interviews	40 healthcare providers	Most providers felt that the optimal age for general discussions about an HIV-infected child’s health should happen around age six, but that specific discussions regarding HIV infection should be delayed to a median of 10 years. Most providers said that primary caregivers were the most appropriate individuals to lead disclosure discussions, but acknowledged that caregivers require support from healthcare providers.
